# The interplay between leaders’ personality traits and mentoring quality and their impact on mentees’ job satisfaction and job performance

**DOI:** 10.3389/fpsyg.2022.937470

**Published:** 2022-11-11

**Authors:** Kalpina Kumari, Salima Barkat Ali, Masooma Batool, Lucian-Ionel Cioca, Jawad Abbas

**Affiliations:** ^1^Faculty of Institute of Business and Health Management, Jinnah Sindh Medical University, Karachi, Pakistan; ^2^Department of Psychology, Iqra University, Karachi, Sindh, Pakistan; ^3^Faculty of Education, Virtual University, Lahore, Pakistan; ^4^Department of Industrial Engineering and Management, Faculty of Engineering, Lucian Blaga University of Sibiu, Sibiu, Romania; ^5^Faculty of Management Sciences, University of Central Punjab, Lahore, Punjab, Pakistan

**Keywords:** leaders’ FFM personality traits, leaders’ mentoring quality, mentees’ job satisfaction, mentees’ job performance, higher education institutions

## Abstract

This study focuses on examining the role of leaders’ Five-Factor Model (FFM) personality traits in their mentoring quality and mentees’ job satisfaction. It has also examined how leaders’ mentoring quality impacts mentees’ job satisfaction, leading to their job performance at the workplace. The study used an explanatory research methodology to determine the cause-and-effect relationship between mentors’ FFM personality characteristics, mentoring quality, and mentees’ job satisfaction and job performance. The study was based on path-goal theory and the Big Five-Factor Model of personality characteristics, and a questionnaire was utilized to collect information on the model’s constructs. Following the non-probability convenience sampling technique, the empirical data were collected from the academic and non-academic staff of public and private higher education institutions (HEIs) located within Pakistan on five-point Likert scale. The proposed hypotheses were tested by using PLS software. Four main conclusions were derived from this study. First, the leaders’ openness to new experiences, agreeableness, and emotional stability substantially influenced the mentees’ job satisfaction. Surprisingly, the leaders’ conscientiousness and extraversion qualities did not affect the job satisfaction of the mentees. Second, the findings demonstrated that the openness to experience, conscientiousness, and extroversion has a considerable influence on leaders’ mentoring quality, but agreeableness and emotional stability have a negligible impact. Third, the mentoring quality of the leader had a substantial effect on the job satisfaction and work performance of the mentees. Fourth, this study confirmed the belief that mentees’ job satisfaction has a favorable influence on their job performance within the context of Pakistan’s educational sector. The current study’s findings provided valuable insights to the educational institutions about which personality traits they need to foster in their leaders, making them an excellent leader to enhance their mentees’ job satisfaction and job performance within their organizational settings.

## Introduction

The service industry has recently emerged as one of the most essential and fastest-growing segments of the global economy. In first-world countries, the services sector accounts for roughly 70% of GDP. The service industry is also advancing rapidly in high-opportunity and low-income countries (Owusu et al., 2020). The service sector’s share in Pakistan’s economy has also grown steadily over the past few decades. It is estimated that 61.68% of Pakistan’s GDP is generated by the services sector, accounting for over one-third of the country’s workforce (Javed and Ilyas, 2018).

Considering the high scope of growth in organizations providing knowledge and skill-based facilities such as educational institutions, it seems worth exploring a promising mechanism of education advancement called mentoring relations in these organizations. Most definitions of the mentoring state that it is an activity in which someone with more expertise helps and supports someone with less expertise to advance their professional careers (Garg et al., 2021). There are two key roles that mentors play in their relationships with protegees, according to Chaudhuri et al. (2021). One is the traditional role that is offering professional support that includes actively participating in the mentee’s development by providing opportunities and challenges to grow and prosper in his career. At the same time, the other is to provide psychosocial support. Krasikova and LeBreton (2012) also have considered two critical functions of the mentoring relationship and defined that the mentor is responsible for teaching mentees explicit and implicit lessons about professional development and overall work–life balance (Arora, 2020).

Additionally, a large body of research confirms that mentoring has a significant impact on the success of educational institutions and the lives of the people who work there (Hakro and Mathew, 2020; Cristofaro et al., 2021). Mentoring programs in organizations have been shown to improve employee performance, job satisfaction, and overall business productivity, among other benefits (Dahlberg et al., 2019; Maloni et al., 2019). Nevertheless, doing righteousness with these dual functions and keeping the balance between them is not an easy task for mentors. However, the most difficult challenge may be merging professional assistance with other areas such as faith, family, and community (Gupta and Gupta, 2021).

Furthermore, several studies have shown the importance of employee commitment and satisfaction in a company’s ability to succeed. This has led to an increased focus on JS as a critical corporate goal in recent years. Many elements have been demonstrated to influence employee JS, including working conditions, supervision, policy and administration, progression, remuneration, interpersonal connections, recognition, and empowerment. However, recent research has shown that the leader’s personality traits significantly impact employees’ JS at the workplace (Kiarie et al., 2017). Park and Johnson (2019) also explained that the quality of the leader–employee relationship influences employee JS, and employees are more likely to be content and comfortable when they feel supported by their leaders.

Moreover, working with a boss who is unsupportive and whose behavior is negative can cause stress for employees. Employee productivity suffers, absenteeism rises, and the turnover rate can be pretty high when the leader–employee relationship is not really good (Asrar-ul-Haq and Anjum, 2020). Therefore, leaders’ personality qualities may be a determining element in an organization’s ability to satisfy personnel, as well as having a substantial impact on how leaders interact with, think about, feel about, view, and even respond to others (Saad et al., 2018; Arendt et al., 2019). As a result of mounting evidence showing the impact of leadership personality traits on employees’ productivity, performance, and satisfaction, organizations are turning their attention to their leaders’ ability to lead, preferred style, and competence (Anastasiadou, 2019).

However, while focusing on the importance of Five-Factor Model (FFM) personality traits, Kiarie et al. (2017) identified the gap that most of the literature talks about the mentees’ personality traits (Arora, 2020) and their impact on various employee-related outcomes (Judge et al., 2000). On the other hand, very rare studies have focused particularly on how mentors’ FFM personality can influence their mentees’ JS and lead to their performance in the workplace (Kiarie et al., 2017). Looking at such previous studies, this was found as a potential gap that needs to be addressed. Therefore, based on the understanding of the dynamic role of the mentor and the lack of empirical studies that used any formal personality test to identify such personality traits that can help the mentor in performing his diversified and challenging role in Pakistani industries, we became interested in exploring if the mentor has a specific personality trait that naturally facilitates him in fostering high-quality mentoring relationship, which eventually can lead toward his mentees’ JS and JP at the workplace.

Moreover, on the other hand, Western service industries are more commonly known for using personality tests to match mentors and mentees. Myers–Briggs Type Indicator (MBTI) personality test is used in around 1,000 empirical research studies, and over 3 million people take this test every year, according to the Center for Applications of Psychological Type (CAPT) (Kahle-Piasecki, 2011). As a result, the research’s findings and conclusions will add significantly to the body of knowledge about the Big FFM of personality traits and their impact on mentees’ JS and JP in the workplace.

Therefore, based on the areas mentioned above of exploration, the overarching aim of this study is to examine the role of mentors’ FFM personality traits in their mentoring quality and mentees’ job satisfaction (JS). It is also examined does mentoring quality impacts mentees’ job satisfaction (JS), leading to their job performance (JP) at the workplace.

## Literature review and conceptual model

### Foundation and hypotheses development

This study’s research model is based on a combination of two theories. The first is Robert House’s path-goal theory, and the second is the Big Five Theory of Personality. According to the path-goal theory, there is a link between a leader’s behavior and the motivation, satisfaction, and performance of employees at work (Alanazi et al., 2013). According to this principle, a leader should also be involved in behaviors that enhance a mentee’s strengths and compensate for their weaknesses. It argues that if a leader helps subordinates in finding the best path to achieve goals that are cognizant of organizational goals, and then employees remain satisfied and remain connected and committed to the organization’s vision. In this study, based on Path-goal theory, it is assumed that the overall personality of the mentor, which includes the mentor’s thoughts, feelings, and behaviors, will have a significant impact on the mentees’ job satisfaction and performance at work.

Additionally, the Big Five Factors of Personality theory is based on the Five-Factor Model (FFM) of personality traits, i.e., openness to experience, conscientiousness, extraversion, agreeableness, and emotional stability. Openness refers to the extent of cultural interests, fantasy, and creativity. It is reflected in the intellect. Conscientiousness is manifested by discipline, an organized approach, and an orientation toward achievement. Extraversion trait is displayed in talkativeness, sociability, assertiveness, and self-confidence. Agreeableness is demonstrated by the help provided to others and a sympathetic attitude toward others. Lastly, the degree of negative emotions, such as anger, anxiety, and despair, which imply emotional instability, is neuroticism (McCrae and Costa, 2013). According to Judge et al. (2000), all the defined personality traits have the most evident links with work satisfaction.

### Mentors’ personality traits and mentees’ job satisfaction

Various psychologists have attempted to define personality, but Mayer (2007) asserted that there is a general agreement among all the definitions given by psychologists at different points of time, and he went on to say that personality is a collection of pieces that are structured, grow, and manifest in a person’s behaviors (Mayer, 2007). According to Larsen et al. (2005), the collection of structured and relatively long-lasting psychological qualities and systems that impact an individual’s interactions with and adaptations to the intrapsychic, physical, and social contexts is referred to as personality (Schmitt et al., 2007). The Big Five model has been widely used and replicated across geographic and cultural contexts (Kiarie et al., 2017).

Literature review reveals that a considerable number of researchers have investigated how mentees’ personality traits can influence their job satisfaction and other related outcomes at the workplace (Judge et al., 2000; Goldner, 2016). In addition, Barrick and Mount (1991) investigated the impact of employees’ “Big Five” personality dimensions (openness to experience, conscientiousness, extraversion, agreeableness, and emotional stability) on three job performance criteria (job proficiency, training proficiency, and personnel data) for five occupational groups (professionals, police, managers, sales, and skilled/semi-skilled). Findings revealed a strong correlation between conscientiousness as an aspect of personality and all of the job performance criteria across all occupational categories. Arora (2020) also investigated the mediating effect of mentoring functions on the relationship between FFM personality traits and occupational commitment and proposed the significant impact of mentees’ FFM personality traits on their occupational commitment.

However, very rare studies were found during the literature review that explored the influence of mentors’ personality traits on their mentees’ job satisfaction. Kiarie et al. (2017) used an explanatory study methodology to establish a cause–effect link between a leader’s personality qualities and employee work satisfaction. The hypotheses were investigated using multiple regression equation models. Extraversion, openness to new experiences, emotional stability, conscientiousness, and agreeableness were found to be effective ways for leaders to improve their workers’ job satisfaction at work (McCrae et al., 2002). Therefore, to study the given relationship between mentors’ personality traits and mentees’ job satisfaction in more depth, the following hypotheses have been developed:

H1a: Mentors’ openness to new experiences significantly affects mentees’ job satisfaction.

H1b: Mentors’ conscientiousness significantly affects mentees’ job satisfaction.

H1c: Mentors’ extraversion significantly affects mentees’ job satisfaction.

H1d: Mentors’ agreeableness significantly affects mentees’ job satisfaction.

H1e: Mentors’ Emotional Stability significantly affects mentees’ job satisfaction.

### Mentors’ personality traits and mentors’ mentoring quality

Studies have shown that mentoring effectively improves work performance and career advancement (Carter and Youssef-Morgan, 2019). While the usefulness and quality of mentoring depend partly on the mentors’ abilities, little study has been done on mentors’ personality traits (Younginer and Elledge, 2021). Existing research has looked at three levels of individual mentor traits—demographic, experience, and personality—and their impact on a mentor’s readiness to be a good mentor (Broshears, 2021). Age, gender, educational level, and experience as a mentor or protege have all been linked to their desire to be a good mentor.

Furthermore, Noypa et al. (2021) discovered that having a prosocial personality predicted the willingness to mentor others, while other researchers favored locus of control (Ekanayaka, 2021) and upward striving (Diaz, 2021) as personality-based motivators of quality mentoring activity. Researchers in personality and industrial psychology have now universally adopted the five-factor model of personality. Extroversion, conscientiousness, agreeableness, neuroticism, and openness to new experiences are all part of it (Wulf et al., 2021). The five-factor model has been shown to be accurate in predicting job performance (Nikbin et al., 2021), motivation (Diaz, 2021), leadership (Yip and Walker, 2021), and workplace deviance (Colbert et al., 2004). However, research into the influence of the five-factor model on the professional mentoring process has also begun. Niehoff (2006) examined the mentors’ personality traits to see how well they predicted the theoretical and statistical relationships between personality, as defined by the five-factor model, and professional mentoring of skilled junior employees, interns, and students. According to the findings, the level of engagement as a mentor was positively connected with extroversion, conscientiousness, and openness to experience. These findings imply that a mentor’s personality attributes may have a more substantial impact on their engagement as a mentor. Because mentoring entails active participation in an environment that necessitates social, task, and idea-related talents, thus people who are extroverted, conscientious, and open to new experiences are more likely to feel at ease to perform their role of mentoring more effectively. Therefore, to explore the relationship between mentors’ personality traits and their level of quality in mentoring process in more depth, the following hypotheses have been proposed:

H2a: Mentors’ openness to new experiences significantly affects mentors’ mentoring quality.

H2b: Mentors’ conscientiousness significantly affects mentors’ mentoring quality.

H2c: Mentors’ extraversion significantly affects mentors’ mentoring quality.

H2d: Mentors’ agreeableness significantly affects mentors’ mentoring quality.

H2e: Mentors’ Emotional Stability significantly affects mentors’ mentoring quality.

### Mentors’ mentoring quality and mentees’ job satisfaction

Job satisfaction is a positive emotional state that results from assessing one’s work situation (Amin, 2021). Workplace satisfaction is correlated with and measured by the following factors: work, promotions, compensation, supervision, coworkers, and general feelings about the job (Lahat and Marthanti, 2021). To improve working conditions, lower absenteeism, increase retention, provide adequate customer service, and attract qualified professionals, it is critical to research the factors that predict job satisfaction (Monroe et al., 2021). Additionally, a mentor is typically a more senior employee who guides a junior-level protégé (Hu et al., 2022), and hence, mentoring is one of the critical roles that a leader holds for the career progress of his employees. Mentoring, on the other hand, can be defined as a process in which one (usually younger) individual (the mentee) is guided in the development and reexamination of their ideas, learning, and personal and professional development by an experienced, highly regarded, and empathetic person (the mentor) (Goldhaber et al., 2022).

Studies have found that mentoring increases: job satisfaction (Jiang et al., 2020); intentions to stay on the job (Jin et al., 2019), organizational socialization (Wang et al., 2018), salaries and promotions of employees (Alston, 2021); and career outcomes in general (Bredella et al., 2021). According to Abraham et al. (2021), mentoring is the leading factor among all other factors that influence employees’ job satisfaction at the workplace. Therefore, it can be predicted that if the leader plays the role of mentor and nurtures the mentee, it can bring positive results to an organization’s workforce. Similarly, one of the studies conducted by Mitchell (2021) on new hire nurse practitioners at the plastic surgery department concluded that new nurse practitioners affirmed that mentoring increases their job satisfaction and is a crucial factor in staying at the organization for a more extended period. Another study conducted by Mehra and Tharakan (2020) proposed that mentoring functions performed by supervisors and coworkers bring better results concerning mentee job satisfaction than those fulfilled by the assigned formal mentor from the upper level in the organizational hierarchy. As a result of the above debate, the following hypothesis has been proposed:

H3: Mentors’ mentoring quality significantly affects mentees’ job satisfaction.

### Mentors’ mentoring quality and mentees’ job performance

One of the essential work relationships that can serve as a platform for personal learning is mentoring (Neupane, 2015). Individuals learn a lot through their contacts with others, especially those from various backgrounds, who have different knowledge and have more experience in the company. Mentoring mechanisms/functions boost mentees’ personal development (Abiddin and Hassan, 2012) by offering informative feedback on diverse activities, which improve mentees’ work performance (Jyoti and Sharma, 2017). Positive mentorship makes a mentee feel secure to ask questions and take on complex projects, and the mentor, in turn, actively listens and invites mentees to discuss various work-related issues, which aids in developing the mentee’s learning capacity (Weiss and Merrigan, 2021). This increase in learning capability and changes in behaviors makes a mentee do their job better (Hsieh and Huang, 2018). Pan et al. (2011) looked at the function of personal learning in mediating the relationship between supervisory mentoring and career success. Mentoring has been shown to alter a protégé’s behavioral (personal learning) reactions to work, resulting in a more favorable work experience (performance).

Further, Lapointe and Vandenberghe (2017) also revealed that mentors are a significant resource for learning organizations, and mentoring (vocational assistance) is positively associated with personal learning. A mentor’s mentoring support helps the protégé to have a better grasp of the job environment by acquiring skills, which may lead to less uncertainty about the expectations connected with their responsibilities in the company. It enables the mentee to enhance job-related abilities (Yang et al., 2021). Additionally, mentoring is favorably associated with mentees’ learning, which influences mentees’ job performance, according to Chatterjee et al. (2021). Mentoring is also a trust-based relationship between two persons in which the mentor provides continuing support and growth opportunities to the mentee (Garg et al., 2021). As a result, a good connection enhances the frequency of engagement and skill learning, allowing people to perform better (Ehinola and Akomolafe, 2022). In a high-quality mentor–protégé relationship, the mentor addresses current difficulties relating to the mentees’ work and provides insights on organizational work styles, informal networks, and obstacles and possibilities, all of which help the mentee to improve his or her job performance. Based on the above debate, the following hypothesis has been established:

H4: Mentors’ mentoring quality significantly affects mentees’ job performance.

### Mentees’ job satisfaction and mentees’ job performance

Job satisfaction is an emotional variable resulting from evaluating an individual’s work experience (Yuen et al., 2018). In another way, job satisfaction refers to how much individuals like their work (Amin, 2021). The principle of equity can account for job happiness. The equity idea refers to the balance between an employee’s contribution and output in the workplace, and the employee gets demotivated or unsatisfied if their input is not appropriately paid compared to someone doing similar work (Ahmad et al., 2021). Furthermore, performance is what the company engages you to do, which must be done successfully (Asbari et al., 2021). It also refers to how well an individual performs their job duties (Torlak and Kuzey, 2019).

However, examining the link between work satisfaction and job performance in an organizational behavior eventually leads to corporate performance. It is necessary to assess the relationship between job satisfaction and job performance due to highly pleased employees outperforming their unsatisfied counterparts (Yuen et al., 2018). According to several researchers, satisfied workers are more likely to be active at work, resulting in low absenteeism, making fewer mistakes, being more productive, and having excellent intentions to stay with the company (Siengthai and Pila-Ngarm, 2016). According to a meta-analysis, job satisfaction shows a favorable relationship with employee performance (Inuwa, 2016). According to da Cruz Carvalho et al. (2020), as an employee’s degree of satisfaction rises, so will the employee’s level of performance (Ones et al., 1994). Based on the above debate, the following hypothesis has been established:

H5: Mentees’ job satisfaction significantly affects their job performance.

## Research methodology

### Research approach and strategy

This study is founded on deductive assumptions, which are popular among positivist philosophers. The study of personality traits, coaching and mentoring from supervisors, and their influence on employee happiness and performance at work begins with existing ideas, models, and literature on the subject of personality traits and employees’ mentoring at the workplace. Furthermore, this study used a survey technique, commonly associated with deductive reasoning and a popular research strategy in management and business studies. This method is commonly used since it allows the cost-effective collection of enormous amounts of data from a significant population (Saunders et al., 2018).

### Target population

**FIGURE 1:**
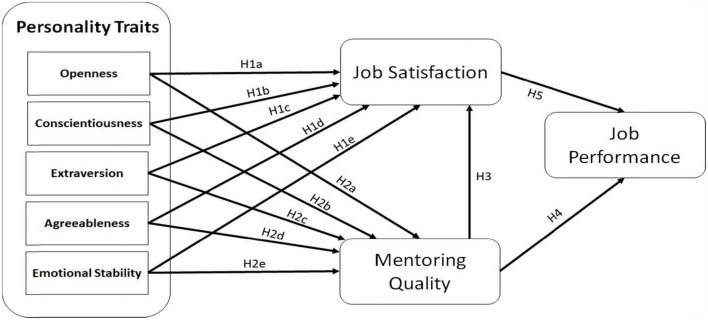
Conceptual framework.

Since the current study focuses on educational institutions, the target population comprises academic and non-academic personnel at public and private higher education institutions in Pakistan’s cities of Karachi, Lahore, and Islamabad. Considering the Higher Education Commission (HEC) of Pakistan as the regulatory body for HEIs located within the country, only those institutions that are being registered with it were approached (see Figure 1).

### Sampling

A research project’s sample size is also a topic of discussion. While some writers argue that higher sample sizes can provide a better representation of the population (Zikmund et al., 2013), others believe that an unnecessarily big sample can lead to the type-II error problem (Sekaran and Bougie, 2016). However, the norm for good research is from 30 to 500 samples or 10 times or more the number of items for variables such as independent, dependent, mediating, moderating, and control factors when deciding on sample size (Sekaran and Bougie, 2003). In this context, the authors circulated 731 questionnaires to the employees of HEIs, with the expectation of receiving fewer than 500 responses, which they considered to be a sufficient sample size according to Sekaran and Bougie (2016). The writers used a variety of approaches to deal with the problem of social desirability. For instance, the questionnaire’s random presentation of a variable’s items. This was done in order to disrupt the respondents’ expected order of answering (Booth-Kewley et al., 2007). The authors also noted that it was crucial for respondents to react appropriately in order to preserve the quality of the results that were drawn from their responses (Ahmad et al., 2021). Additionally, the responders received reassurances that the data they submitted to the authors would be treated confidentially (Kong et al., 2021).

Since it was impossible to get accurate information about the total number of academic and non-academic staff working in the HEIs of Pakistan, the authors preferred to follow a convenience-based sampling technique to collect the data. The given sampling technique has also been considered an ideal approach when we don’t have access to the whole population (Malhotra and Dash, 2011).

Employees having a minimum of 1 year of work experience were surveyed to guarantee that they had spent enough time in the business to complete the questionnaire on their mentor’s personality traits, mentoring quality, and job satisfaction and performance at the workplace. Demographic variables in the study included the name of respondents, gender, age, education, experience, and nature of work. For further detailed information on respondents’ demographic, see Table 1.

**TABLE 1:** Demographic characteristics of respondents (*N* = 314).

Particulars	Description	Values	%
Gender	Male	191	60.83%
	Female	123	39.17%
Age	20–30	65	20.7%
	31–40	124	39.49%
	41–50	99	31.53%
	50 +	26	8.28%
Education	Undergraduate	15	4.78%
	Graduate	114	36.31%
	Postgraduate	158	50.32%
	Ph.D. or Certification	27	8.60%
Years of experience	<2 years	17	5.41%
	2–5 years	96	30.57%
	6–10 years	175	55.73%
	More than 10 years	26	8.28%
Nature of work	Academic Staff	208	66%
	Non-academic staff	106	34%

### Data collection method

The data for the current study were gathered using an empirical method. During the COVID-19 era, data were collected from the academic and non-academic staff of public and private HEIs through a structured questionnaire administered by a web-based platform, namely, Google Forms.

Standardized self-administered questionnaires were distributed through email to 731 respondents (due to COVID-19, it was difficult to approach the employees individually, and outsiders were also not allowed to enter the company). Only 235 completed questionnaires were received after 3 weeks on the first hand. To gather information from the remaining participants, the soft reminder calls through email were made again, and 85 more responses were received effectively. After removing unusable questionnaires (excluded during the data screening process due to incomplete or unviable responses), 314 useable questionnaires were obtained with a response rate of 42.5%. Finally, 314 completed responses were analyzed with structural equation modeling (SEM) using partial least squares software (Smart PLS v. 3). The process of data collection took around 10–12 weeks.

### Description of measures

The items were selected from validated questionnaires used in previous research studies. In order to measure the mentors’ personality traits, 10 items scale was drawn from the research of Gosling et al. (2003). Examples of sample items included “*I see my supervisor as extraverted, enthusiastic*” *and “I see my supervisor as reserved, quiet*.” Mentoring quality was measured by using the instrument of Dreher and Ash (1990), which consisted of 04 items scale. Examples of sample items included “*I have a mentor who gives or recommends me for challenging assignments that present opportunities to learn new skills” and “I have a mentor who conveys feelings of respect for me as an individual*.” Additionally, mentees’ perception of their job satisfaction was measured by using 03 items scale, which was developed by Brayfield and Rothe (1951). Examples of sample items included “*Generally speaking, I am delighted with my job” and “I consider my job to be rather unpleasant*.” Finally, a five-item scale for in-role job performance was adapted to measure mentees’ performance by Podsakoff and MacKenzie (1989). Examples of sample items included *“I always complete the duties specified in my job description” and “I never neglect any aspects of the job that I am obligated to perform.”* The data were collected using a 5-point Likert scale, with 1 indicating strongly disagree and 5 indicating strongly agree.

### Reliability and validity

The authors conducted pilot research to test the questions’ feasibility, clarity, and suitability before undertaking the complete survey, as advised by Hinkin (1998). The pilot study involves data collection from 45 academics and non-academic staff of public and private HEIs. Cronbach’s alpha (α) was used to assess the study’s and research instrument’s internal reliability for the pilot study. The Cronbach alpha for all constructs exceeded the permissible range of 0.7 in the pilot test. For further detailed information, see Table 2.

**TABLE 2:** Reliability analysis.

Variables	Number of items	Cronbach’s alpha
Mentors’ personality traits	10	0.709
Mentoring quality	04	0.801
Mentees’ job satisfaction	03	0.871
Mentees’ job performance	05	0.894

Additionally, before delivering the link to the respondents, the initial questionnaire was produced and approved by academic experts who specialized in human resource management, specifically in the mentoring and counseling arena. They examined the questionnaire’s content and the extent to which it was likely to measure the research variables, i.e., mentors’ personality traits, mentoring quality, mentees’ job satisfaction, and their job performance at the workplace. However, positive comments were received from all the experts. As a result, no additional adjustments were made to the instrument used in the pilot investigation, and the same was carried forward for the comprehensive study.

The square root value of the average variance explained must be greater than the correlational values among variables for discriminant validity. Table 3 indicates that the proposed requirement is adequately matched, representing the discriminant validity of the studied constructs.

**TABLE 3:** Discriminant validity analysis.

Variable	Pers.	Men. Qu.	Job Sat.	Job Per.
Personality	**0.820**			
Mentoring quality	0.574	**0.836**		
Job satisfaction	0.584	0.645	**0.834**	
Job performance	0.558	0.587	0.583	**0.818**

Pers.: Personality; Men. Qu.: Mentoring Quality; Job Sat.: Job Satisfaction; Job Per.: Job Performance. Bold values indicate the square root of AVE.

## Data analysis and hypotheses testing

Structural equation modeling (SEM) was used to analyze the data. SEM has gained extensive admiration across numerous fields such as human resource management, strategic management, accounting, operations management, management information systems, marketing, supply chain management, hospitality, and tourism (Hair et al., 2017; Cheah et al., 2018). According to Hair et al. (2017), it has high a predictive power to examine complex higher-order models. SEM offers the advantages of examining latent constructs through path analysis and an accentuated explanation of various independent variables while assessing the structural model (Hair et al., 2014). There are two models through which SEM analyzes data. First is the measurement model, which provides information about the relationship between observed and latent variables. The second is a structural model that examines the latent variables’ relationships (Hair et al., 2019). The measurement and structural model analyses adequately complied with suggested values, confirming the fitness of the measurement and structural models (see Table 4).

**TABLE 4:** Analysis of measurement and structural model.

Goodness of fit measures	CMIN/DF^a^	NFI	GFI	AGFI	CFI	TLI	RMSEA	SRMR
Recommended value	≤3	≥0.9^b^	≥0.9^b^	≥0.9^b^	≥0.9^b^	≥0.9^b^	≤0.08^c^	≤0.8^d^
Measurement model	1.668	0.910	0.911	0.902	0.912	0.914	0.033	0.0458
Structural model	1.675	0.921	0.920	0.915	0.920	0.921	0.039	0.0467

^a^Bagozzi and Yi, 1988.

^b^Bentler and Bonett, 1980; McDonald and Marsh, 1990.

^c^Browne and Cudeck, 1992.

^d^Hu and Bentler, 1998.

The proposed hypotheses were examined via SEM. The path analysis indicated a significant positive impact of openness, agreeableness, and emotional stability on mentees’ job satisfaction with beta values of 0.221, 0.241, and 0.269, respectively. Thus, hypotheses H1a, H1d, and H1e are accepted. However, consciousness and extraversion presented positive but insignificant results with 0.144 and 0.139 beta values. Thus, hypotheses H1b and H1c are rejected. Similarly, the analysis of mentors’ personality traits with mentoring quality also indicated mixed results. Openness, consciousness, and extraversion presented significant positive effects with 0.225, 0.368, and 0.256 beta values concerning their relationship with the mentoring quality. Thus, hypotheses H2a, H2b, and H2c are accepted. On the other hand, agreeableness and emotional stability presented insignificant results with 0.149 and 0.155 beta values. Thus, hypotheses H2d and H2e are rejected. Further, the authors examined the impact of mentoring quality on mentees’ job satisfaction which presented 0.382 beta value. This means that mentoring quality is a significant positive predictor of mentees’ job satisfaction. Thus, H3 is also accepted. Similarly, mentoring quality and job satisfaction analysis on mentees’ job performance presented 0.366 and 0.389 *p* values, respectively. Thus, hypotheses H4 and H5 are also accepted (see Table 5).

**TABLE 5:** Examining the hypotheses.

Hypothesis	Constructs	Estimate	Critical ratio	*P*-value	Decision
H_1a_	Open. → Job Satis.	0.221	2.341	0.014	Accepted
H_1b_	Cons. → Job Satis.	0.144	1.447	0.070	**Rejected**
H_1c_	Extr. → Job Satis.	0.139	1.337	0.073	**Rejected**
H_1d_	Agree. → Job Satis.	0.241	2.336	0.018	Accepted
H_1e_	Emo. Sta. → Job Satis.	0.269	2.391	0.021	Accepted
H_2a_	Open. → Ment. Qual.	0.225	2.352	0.012	Accepted
H_2b_	Cons. → Ment. Qual.	0.368	3.860	0.008	Accepted
H_2c_	Extr. → Ment. Qual.	0.256	2.532	0.009	Accepted
H_2d_	Agree. → Ment. Qual.	0.149	1.357	0.069	**Rejected**
H_2e_	Emo. Sta. → Ment. Qual.	0.155	1.478	0.066	**Rejected**
H_3_	Ment. Qual. → Job Sati.	0.382	3.885	0.000	Accepted
H_4_	Ment. Qual. → Job Perf.	0.366	3.784	0.000	Accepted
H_5_	Job Sati. → Job Perf.	0.389	3.984	0.000	Accepted

Open.: Openness; Ment.Qual.: MentoringQuality; Extr.: Extraversion; Agree.: Agreeableness; Emo.Sta.: EmotionalStability; JobSati.: JobSatisfaction; JobPerf.: JobPerformance; Cons.: Conscientiousness. Bold values indicate the square root of AVE.

## Discussion

This study examines the role of mentors’ FFM personality traits in their mentoring quality and mentees’ JS. It has also discussed about how mentor quality impacts mentees’ JS, leading toward their JP at the workplace.

The structural analysis presented a significant positive impact on three of mentors’ personality traits, i.e., openness to experience, agreeableness, and emotional stability, on mentees’ JS. In the current study, it has been found that the more the mentor is inclined toward the trait of openness will lead toward mentees’ JS at the workplace. Aydogmus et al. (2018) also found in their research that openness to change was substantially connected with employee job satisfaction. Similarly, Judge et al. (2000) found a statistically significant association between openness to experience and employee work satisfaction. Kiarie et al. (2017) also reported that openness to experience in mentors’ personalities promotes positive work-related feelings and happiness in their mentees at the workplace.

Similarly, high agreeableness in mentors was found to significantly predict job satisfaction among mentees at the workplace. Assertive and cooperative individuals are more likely to manage a team and improve employee satisfaction. However, because trust and collaboration are two-way streets, employee work satisfaction is determined by their level of trust and cooperation with their leaders. The same findings were reported by Kiarie et al. (2017), who highlighted that agreeableness had the most vital link with employee job satisfaction. It was also claimed that agreeability was linked to thoughtfulness, which makes sense given that agreeable people are sympathetic and empathic. According to Judge et al. (2000), warmth was also associated with employee work satisfaction, as warmth influences a leader’s demonstration of individualized concern.

Furthermore, a significant positive relationship has also been found between mentors’ emotional stability and mentees’ JS at the workplace. Arora and Rangnekar (2016) also reported that emotionally stable mentors can fulfill mentoring responsibility with more resilience and can handle demands and stressors in a more effective way to achieve this role as compared to those senior employees who may have much technical experience in the field but are less equipped in handling stressful situations. Kiarie et al. (2017) also reported that a leader’s emotional stability significantly affects employee job satisfaction.

However, very surprisingly, conscientiousness and extrovert personality traits of mentors were found to be insignificantly impacting mentees’ JS. However, very similar results were also found by Eason et al. (2015), while they explored the role of personality traits in JS among Collegiate Athletic Trainers. Two of the personality traits, i.e., extroversion and conscientiousness, showed a weak positive relationship with JS.

With the help of empirical results, the current study also concluded that to enhance the mentoring quality, mentors need to have the three most important personality traits, i.e., extroversion, conscientiousness, and openness to experience. The outcomes of this study are consistent with generally held beliefs that mentors have some essential attributes that enable them to perform their job of mentoring more effectively. Those who often participate as good mentors, according to Niehoff (2006), are likely to be outgoing, conscientious, and open to new experiences. Such activities need the mentor’s capacity to communicate, which extroverts are more likely to possess than introverts. Mentoring is also a prosocial activity that necessitates the mentor’s commitment to task completion and the development of a positive working relationship with the protégé. More conscientious individuals are more likely to keep such promises than those who are less scrupulous. Finally, the lack of structure in mentoring allows for new viewpoints and problem-solving chances. Individuals that are open to new experiences are likely to be drawn to such circumstances. In terms of personality and leadership, these findings are similar to Judge et al. (2000). Extroversion, conscientiousness, and openness to new experiences were revealed to be powerful predictors of leadership effectiveness and emergence in various contexts. According to Ashton and Lee (2001), the five-factor model’s three features do indeed cluster together. They used the five-factor model and the sixth dimension of honesty in their personality studies. Conscientiousness, extroversion, and openness were all linked to active participation in three areas of endeavor: social, task, and idea-related, according to their findings. Mentoring provides a setting where people are actively involved in all three areas. Individuals with higher levels of this cluster of attributes should be more inclined to gravitate toward mentorship than those with lower traits. However, two of FFM’s personality traits, i.e., agreeableness and emotional stability, were insignificantly impacting mentors’ mentoring quality.

Additionally, the current study reported a positive and significant impact of high-quality mentoring on mentees’ JS at the workplace. The same findings were reported by Kalkavan and Katrinli (2014), who looked at the direct impacts of managerial coaching on employee work performance, job satisfaction, role ambiguity, and satisfaction with managers. The findings revealed that organizational coaching behavior in the insurance industry had a favorable impact on employees’ better understanding of their roles (role clarity), job happiness, career commitment, employee performance at work, and organizational commitment.

The current study also reported a positive and significant impact of high-quality mentoring on mentees’ JP at the workplace. The study’s main finding revealed that employees who believed their bosses had coached them and exhibited excellent coaching behaviors had better job performance as a result. Ali et al. (2018) also discovered that managerial coaching positively predicted employee job performance. Finally, the current study found that mentees’ JS had a favorable and substantial influence on their JP at work. The same findings were reported by Ali et al. (2018), who said that employee work happiness had a beneficial effect on employee job performance. Talukder et al. (2018) have also proven that workplace pleasure is a crucial predictor of improved employee productivity.

### Practical implications

The continuous COVID-19 crisis forces educational institutions to implement stringent measures, resulting in downsizing and increased strain on academics and staff members, as well as the burden of efficiency improvement. Simultaneously, technological advancements not only allow people to be constantly connected and available for work-related tasks (Zawacki-Richter, 2021) but it also necessitates entirely new skills from the workforce, putting more demands and pressure on employees (Baker et al., 2021). The preceding implies that improving employee performance is critical for today’s business success and for organizations to gain a competitive advantage in the ever-competitive business environment. Recently, Al Hemeiri et al. (2021) have confirmed that the extent to which firms can embrace a mentoring culture as a key to employee success will determine their competitive market viability and boost employee performance. Employee mentoring, which includes career assistance, knowledge transfer, and psychological support, is thus recommended for leaders and managers in this field as it has significant positive effects on employees’ performance in achieving their firms’ goals. However, to give high-quality mentoring, this study has found certain personality qualities, i.e., openness to experience, conscientiousness, and extroversion, can assist mentors in improving their mentees’ JS and JP at work. Therefore, to provide effective mentoring to the mentees, educational institutions should first train their mentors to develop the required personality traits, as these training and development schemes can promote future leaders and a capable workforce for the educational sector of Pakistan’s future competitive business environment.

Furthermore, businesses are encouraged to use personality inventories such as the FFM of personality for various objectives, including mentor recruitment and selection. According to the findings of this study, the FFM is a valuable measure for predicting senior employee suitability to fulfill the role of mentor and improve mentees’ JS and workplace performance.

### Limitations and future recommendations

There are at least five potential limitations regarding the outcomes of this study that should be considered while interpreting the results as more research is required.

The first limitation is concerned with designing of the research. We did not evaluate the selected firms’ structure, environment, or cultures. Although each of these traits has the potential to influence one’s role inside an organization, we are unable to explain how these elements influenced our participants’ job satisfaction. Therefore, to grasp an even better understanding of the given phenomena, future researchers are required to examine the role of structure, climate, or cultures in the relationship of mentors’ personality traits, their mentoring quality, and leading mentees’ JS and their JP within the context of the selected organizations.

A second potential limitation is methodology. We used a strictly quantitative approach and only gathered the perception of mentees about their mentors’ personality traits. It would be building on this knowledge and interesting to measure the personality of mentors simultaneously using other than self-report measures and then study the effect of different personality traits on mentoring quality and their mentees’ job satisfaction. The use of a mixed-method approach would also open new horizons to understand the relationship between personality traits and mentoring relationships.

Third, the study’s target population is drawn from a particular component of Pakistan’s educational system. As a result, keep in mind that the findings may not apply to all other Pakistani businesses. As a result, additional segments of other industries might be explored in future research to study the stated link. These areas might include health, defense, and the social sector, among others. In addition, because the public and private sectors have different cultures and work settings, a future study in both sectors should broaden the scope of leadership personality traits.

The study’s fourth restriction is that it is cross-sectional in nature. Because cross-sectional studies cannot establish vital cause-and-effect relationships, future researchers should investigate the impact of mentors’ personality traits over time. Other studies have shown that the time of year can influence personal satisfaction assessments, workload, and balance (Bialowolski et al., 2021). Finally, our findings also serve as a foundation for future evaluations to measure personalities and their relationships to other characteristics such as burnout and work–life conflict.

## Conclusion

Conclusively, finding from the present study, despite several limitations, is of paramount value. The present study highlights four key findings. First, the mentors’ openness to experience, agreeableness, and emotional stability traits displayed a significant impact on mentees’ JS. However, very surprisingly, conscientiousness and extraversion traits of mentors showed an insignificant effect on mentees’ JS. Second, the results show that openness to experience, conscientiousness, and extroversion significantly impact mentoring quality, whereas agreeableness and emotional stability traits showed insignificant impact on mentoring quality. However, third, mentoring quality of mentors significantly impacted mentees’ JS and JP. Fourth, as widely accepted, this study endorsed the significant positive impact of mentees’ JS on their JP within the educational sector of Pakistan. Therefore, the given research has confirmed that certain personality traits proved to enhance mentors’ mentoring quality and ability, and they were also found to positively affect mentees’ JS and their JP. To summarize, this study broadens our understanding of leadership variations in personality, resulting in workers feeling more of a sense of belonging and competence at work, which improves their job performance.

## Data availability statement

The raw data supporting the conclusions of this article will be made available by the authors, without undue reservation.

## Ethics statement

Ethical review and approval was not required for the study on human participants in accordance with the local legislation and institutional requirements. Written informed consent for participation was not required for this study in accordance with the national legislation and the institutional requirements.

## Author contributions

MB and L-IC have made significant contributions to revise and improve the manuscript. All authors contributed to the conceptualization, formal analysis, investigation, methodology, and writing and editing of the original draft, and read and agreed to the published version of the manuscript.
